# Does Mental Health Affect the Decision to Vaccinate Against SARS-CoV-2? A Cross-Sectional Nationwide Study Before the Vaccine Campaign

**DOI:** 10.3389/fpsyt.2022.810529

**Published:** 2022-02-04

**Authors:** Julian Maciaszek, Marta Lenart-Bugla, Dorota Szcześniak, Paweł Gawłowski, Wojciech Borowicz, Błażej Misiak, Joanna Rymaszewska

**Affiliations:** ^1^Department of Psychiatry, Wroclaw Medical University, Wroclaw, Poland; ^2^Department of Emergency Medical Service, Wroclaw Medical University, Wroclaw, Poland; ^3^Department of Pediatric Infectious Diseases, Wroclaw Medical University, Wroclaw, Poland

**Keywords:** COVID-19, SARS-CoV-2, anxiety, mental deterioration, vaccine decision-making

## Abstract

The COVID-19 pandemic generated a sense of threat in the society, leading to social isolation and mental health deterioration. A great deal of hope for the development of herd immunity was placed in preventive vaccinations. The survey, performed before vaccine campaign between September 26-October 27, 2020, during the second wave of the SARS-CoV-2 pandemic in Poland with the Computer Assisted Web Interviews method. The study was partly community based and partly open to the public. Participants were invited to complete the survey using Google forms *via* social media (Facebook, WhatsApp). The survey was also distributed 54 times at the request of interested persons *via* e-mail. Total 1,043 questionnaires were assessed for eligibility and 41 were excluded (13 because of the age under 18, and 28 due to refusal to participate: non-response after sending questionnaire *via* e-mail). Finally 1,001 questionnaires were included to the study and statistical analysis was performed on the basis of the 1,001 responses. The questionnaire consisted of three parts: a sociodemographic survey, a questionnaire assessing the knowledge of the SARS-CoV-2 and the General Health Questionnaire-28. Participants also determined their attitude toward being vaccinated against SARS-CoV-2. The questionnaire was completed by a total of 1,001 participants: 243 people declared that they will not get vaccinated against SARS-CoV-2. Majority of people declaring the willingness to vaccinate were representatives of medical professions, suffering from chronic diseases, with higher values on the total GHQ-28 scale and the subscales: anxiety and insomnia, social dysfunction and somatic dysfunction. Loss of income, difficult access to health care, recognizing the restrictions as excessive and knowledge about COVID-19 were found as significant positive determinants of the reluctance to vaccinate. Greater readiness to vaccinate can be associated with greater certainty about its effectiveness and a hypothetical collectivist attitude. Experiencing anxiety and psychopathological symptoms are risk factors for infection, but can also be conducive to reliance on information about vaccination presented in the media. Reluctance to vaccinate may result from greater awareness of the complexity of the disease, and thus less faith in the effectiveness of vaccines.

## Introduction

Analyzes prepared by the WHO Collaborating Center for Infectious Disease Modeling predicted the effects of the SARS-CoV-2 pandemic at the level of the 1,918 influenza pandemic, killing 50 million people (1). The average mortality rate of SARS-CoV-2 is 2.2%, the Infection Fatality Rate (IFR) ranges from 0.3 to 0.6% (2, 3). To date, over 5 million people have died from COVID-19 worldwide (4). Due to reorganization of the health care system, a reduction in the total number of hospitalizations and planned procedures (5, 6), hospitalizations due to acute coronary syndromes (7, 8) and oncological operations (9) was observed. As a result of these changes, many countries have seen an increase in the number of deaths compared to previous years, also after taking into account those caused by COVID-19 (10). The introduced lockdowns also contributed to the severe economic crisis and an increase in the unemployment in most countries (11).

The COVID-19 pandemic generated a sense of threat in the society, modified lifestyles, leading to social isolation, and thus contributing to a reduction in the quality of life (12). In the course of the pandemic in the general public, symptoms of post-traumatic stress disorder and depression, as well as increased and anger were observed (13–15). In the previous study, analogous to the current one, conducted during the first wave of SARS-CoV-2 in Poland, over 50% of respondents showed at least mild psychopathological symptoms (16). A study by Babicki et al. (17) in the Polish population indicated an equally high prevalence of psychopathological symptoms also during the second wave of the pandemic. The impact of the pandemic on anxiety seems to be particularly important, as confirmed by the study conducted by Greenhawt et al. (18), based on approximately 5,000 respondents whose mean state anxiety score (S-anxiety) was significantly higher than mean trait anxiety score (T-anxiety), with both scores being significantly higher than the previously published standards. The meta-analysis by Bueno-Notivol et al. (19) indicates that the pooled prevalence of depressive symptoms in society during the COVID-19 pandemic is estimated at 25%—approximately seven times greater compared to the average prevalence of pre-pandemic depression, estimated at 3.44%. A study comparing the first and second waves of COVID-19 also confirmed the persistent negative impact of the pandemic on the quality and duration of sleep (20).

A great deal of hope for the development of herd immunity was placed in preventive vaccinations. So far, on November 4, 2021, 39% of the world's population was fully vaccinated against SARS-CoV-2. Individual countries differ significantly depending on the number of complete vaccinations, e.g., USA 57%, Israel 65%, Germany 66%, Poland 53% and Russia 33% (21).

So far, only individual studies examining the factors influencing the decision to vaccinate have been published. Due to the importance of the topic, this original survey is aimed to identify the relationship between the decision to vaccinate and demographic factors, mental health measured with the standardized GHQ-28 questionnaire and pandemic-related factors. We hypothesize that the presence of psychopathological symptoms, as well as the level of knowledge on SARS-CoV-2 determine the willingness to be vaccinated.

## Materials and Methods

The survey was performed from September 26, 2020 to October 27, 2020, during the second wave of the SARS-CoV-2 pandemic in Poland. At that time, there was a sharp increase in the number of reported positive test results and, due to the epidemiological situation, additional restrictions were introduced, such as the obligation to cover the mouth and nose in public spaces (22).

**Figure 1:**
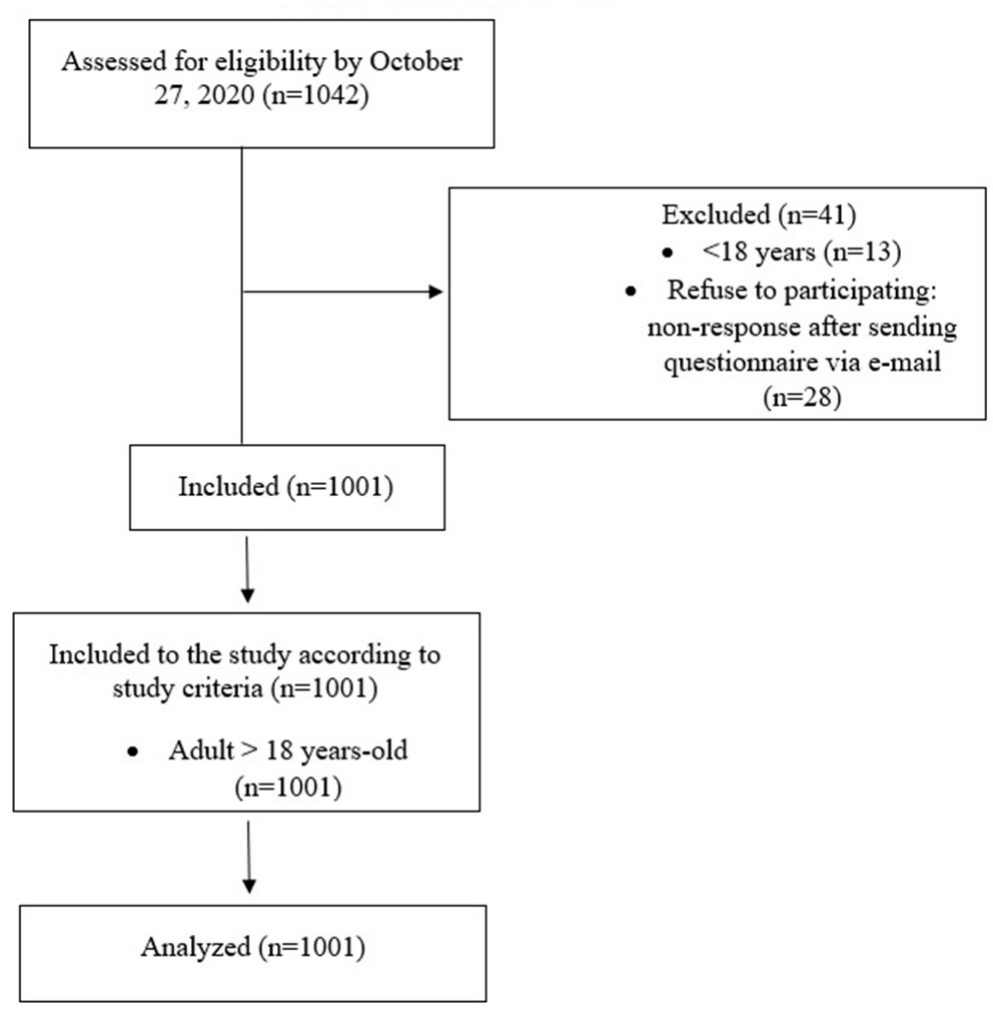
STROBE flow chart.STROBE.

At the time of data collection, no SARS-CoV-2 vaccines were available and no reports of their efficacy were published. The questionnaires were obtained using the Computer Assisted Web Interviews (CAWI) method, which is currently one of the most popular and fastest growing survey methods. Thanks to the feeling of anonymity and the opportunity to participate in the survey at a time convenient for the respondent, it allows to collect more reliable data. The manuscript was formulated based on STROBE Statement—cross-sectional reporting checklist (23) and the protocol was described in the STROBE flow chart (Figure 1). A priori analysis performed using G^*^ Power software (24) revealed that to detect a correlation with *r* = 0.01 and power of 0.95, the calculated sample size was 595. Due to the potential non-response, questionnaires were sent to more participants. The study was partly community based and partly open to the public. Participants were invited to complete the survey using Google forms *via* social media (Facebook, WhatsApp) and information about the survey was also posted on the website of the Department of Psychiatry of the Wroclaw Medical University. In the case of people willing to complete the survey who do not use social media, the survey was also distributed 54 times at the request of interested persons *via* e-mail. The questionnaire was fully anonymous, aimed at people aged 18 and over, and only fully completed questionnaires were analyzed. Total 1,043 questionnaires were assessed for eligibility and 41 were excluded (13 because of the age under 18, and 28 due to refusal to participate: non-response after sending questionnaire *via* e-mail). Finally 1,001 questionnaires were included to the study and statistical analysis was performed on the basis of the 1,001 responses.

All participants gave their informed consent to participate in the survey. The study procedure was approved by the Ethics Committee of the Medical University of Wroclaw (Poland, no 188/2020) and performed in accordance with the principles of the Helsinki Declaration.

The study consisted of three parts: a sociodemographic survey, a questionnaire assessing the knowledge of the SARS-CoV-2 pandemic and the General Health Questionnaire-28 (GHQ-28). Participants also determined their attitude toward being vaccinated against SARS-CoV-2, choosing from the following responses: (a) “I will definitely not get vaccinated against SARS-CoV-2”; (b) “I would make a decision based on the ratio of vaccine efficacy to the observed side effects”; (c) “I will definitely get vaccinated against SARS-CoV-2”.

The sociodemographic survey included questions about sex, age, place of residence, education, the presence of chronic diseases and the use of psychological or psychiatric care. This section also included questions about the impact of lockdown on income, access to medical care, frequency of tracking the epidemiological situation, main sources of knowledge about the SARS-CoV-2 pandemic, and assessment of the extent of the lockdown. The full sociodemographic survey is available in the Supplementary Table S1.

The original questionnaire of knowledge about COVID-19 included 10 questions, for each correct answer, participants could get one point. Question number 1 regarded the current definition of a pandemic, questions 2,3,4,6 concerned the virulence and course of SARS-CoV-2 infection, questions 5,7,8 concerned the measurable effects of the pandemic, and questions 9 and 10 regarded knowledge of personal protective equipment. The detailed questionnaire of knowledge about COVID-19 is available in the Supplementary Table S2.

The number of correct answers was included as the measure of knowledge (Supplementary Table S2). The Cronbach's alpha in the total sample was 0.716, indicating acceptable internal consistency. In our previous study, we presented the relationship between mental health and knowledge of SARS-CoV-2 (25).

The GHQ-28 is a questionnaire that assesses the prevalence of psychopathological symptoms in the general population. It consists of 28 questions divided into four categories of symptoms: severe depression (items 6, 19, 20, 21, 22, 23, 24), anxiety and insomnia (items 2, 7, 9, 13, 15, 17, 18), disorders of social functions (items 5, 10, 11, 25, 26, 27, 28) and somatic symptoms (items 1, 3, 4, 8, 12, 14, 16) (26, 27). The points range from 0 to 84 points, with a higher score indicates greater psychopathology in the mental picture. The cut-off point for clinical significance was set at 24 points, as described by Makowska and Merecz (27).

Only fully completed questionnaires were used for statistical analysis. The following procedure was used: anonymous responses received *via* Google Forms were identified by code numbers, checked for completeness and submitted for further analysis.

The Mann-Whitney U test or *t*-test, respectively, were used to compare participants for continuous values. The Shapiro-Wilk test was used to evaluate the normal distribution. The chi-square test was used to assess the differences between the groups in terms of categorical variables. Additionally, a binary logistic regression was performed. Reluctance to vaccinate against SARS-CoV-2 was defined as the dependent variable. The independent variables were the factors that significantly differentiated the anti-vaccination and pro-vaccination groups with respect to the bivariate comparison. Three models differing from the independent variables were created to determine the model with the highest value of Negelkere's R2 that most fully described the effect on the dependent variable. The higher Negelkere's R2 value, the greater the proportion of variance 'explained' by the regression model makes it a useful measure of the success of predicting a dependent variable from independent variables.

In the first step, we took into account the psychopathology described in the GHQ-28 subscales. Next, we added sociodemographic factors. Finally, we extended the previous models to include factors related to the pandemic, considering the level and source of knowledge about COVID-19, as well as the impact of lockdown and attitudes to the introduced restrictions.

The results were considered significant if the *p-*value was < 0.05. All analyzes were performed in SPSS (IBM SPSS Statistics for Windows).

## Results

### General Characteristics

In the current study, 1,001 responses were collected. Among the respondents, 243 people (24%) declared “I will definitely not get vaccinated against SARS-CoV-2”, 574 people (57%) declared “I would make a decision based on the ratio of vaccine effectiveness to the observed side effects”, and 184 people (18%) declared “I will definitely get vaccinated against SARS-CoV-2”. Table 1 presents the characteristics of the study group taking into account gender. Almost 75% of the respondents were women, the average age was 38 years (standard deviation [SD]: 14.6, range 18–83), 90% lived in the city, almost 76% had higher education, almost 48% worked in the medical profession, 21% suffered from chronic somatic diseases and 17% received psychiatric or psychological care (Table 1). Using the GHQ-28 scale showed that 39% of all respondents obtained more than 24 points, which suggests the presence of clinically relevant psychopathological symptoms. The mean GHQ-total score was 22.86 (SD: 12.9 points, range: 1–75). Over 27% of respondents reported losing income as a result of the lockdown, and over 45% reported difficult access to healthcare during the pandemic. In the study sample, 42% monitored the epidemiological situation every day, over 34% described the previously introduced lockdown as excessive, and 38% indicated the mainstream media as the main source of knowledge about the COVID-19 pandemic. In the questionnaire of knowledge about COVID-19 the average score was 6.0 points (SD: 2.1, range: 0–10). Compared to men in the study group, women were significantly more likely to work in health care, had a higher severity of social dysfunction and somatic symptoms, more often than men indicated limited access to health care, more often indicated the daily monitoring of the epidemic situation and more often relied on the mass media as the main source of information about the pandemic. Men in the study group achieved significantly higher results in the COVID-19 questionnaire and significantly more often indicated an excessive range of introduced lockdowns.

**Table 1:** General characteristics of total sample. *n* (%) or mean ± standard deviation.

	**Total** ***n =* 1001**	**Women** ***n =* 750 (74.85%)**	**Men** ***n =* 251 (25.15%)**	***p-*value**
Age, years	38.36 ± 14.62	38.17 ± 14.19	38.91 ± 15.82	0.867
Place of residence (urban)	901 (90.01%)	675 (90%)	226 (90.04%)	0.971
Education level (higher education)	759 (75.82%)	576 (76.80%)	183 (72.91%)	0.276
Occupation (medical profession)	479 (47.85%)	382 (50.93%)	97 (38.65%)	**0.031**
Chronic diseases (yes)	210 (20.98%)	167 (22.27%)	43 (17.13%)	0.225
Psychiatric or psychological care	172 (17.18%)	138 (18.40%)	34 (13.55%)	0.179
GHQ-28 positive scoring	394 (39.36%)	318 (42.40%)	76 (30.28%)	<0.000
GHQ-28—Total score	22.86 ± 12.9	23.72 ± 13.34	20.29 ± 11.04	0.692
GHQ-28—somatic symptoms	5.66 ± 3.8	6.01 ± 3.89	4.65 ± 3.28	**0.000**
GHQ-28—anxiety and insomnia	6.58 ± 4.7	6.95 ± 4.76	5.46 ± 4.18	0.105
GHQ-28—social dysfunction	7.66 ± 2.9	7.72 ± 3.01	7.46 ± 2.50	**0.019**
GHQ-28—severe depression	2.96 ± 3.8	3.04 ± 3.80	2.72 ± 3.57	0.168
Vaccination (anti-vaccination)	243 (24.28%)	182 (24.27%)	61 (24.30%)	0.645
Loss of income	277 (27.67%)	206 (27.47%)	71 (28.29%)	0.979
Difficulty in accessing heathcare	457 (45.65%)	357 (47.60%)	100 (39.84%)	**0.029**
Daily tracking of the epidemiological situation	424 (42.36%)	320 (42.67%)	104 (41.43%)	**0.000**
Opinion: the applied lockdown was excessive	348 (34.77%)	238 (31.73%)	110 (43.82%)	**0.020**
Mass media as main source of information	382 (38.16%)	345 (46.00%)	37 (14.74%)	**0.037**
Knowledge about SARS-CoV-2: number of correct answers	6.0 ± 2.1	5.85 ± 2.12	6.48 ± 2.12	**0.000**

*Data expressed as n (%) or mean (SD). Significant effects (p < 0.05) are marked in bold*.

### Bivariate Comparisons

Table 2 shows the comparison of the two groups in terms of the declared willingness to vaccinate. The first group included people definitely reluctant to vaccination (anti-vaccination), the second group included the remaining people considering or already decided to vaccinate (pro-vaccination). The pro-vaccination attitude was significantly more often observed among representatives of medical professions and people with chronic diseases. People declaring the willingness to vaccinate obtained significantly higher values on the GHQ-28 scale, both in relation to the total results and the subscales: anxiety and insomnia, social dysfunction and somatic dysfunction. Nearly 33% of people reluctant to get vaccinated and over 41% of those willing to vaccinate experienced significant clinical psychopathological symptoms. Respondents from the pro-vaccination group significantly more often confirmed the daily monitoring of the epidemiological situation and more often indicated the mass media as the main source of information about the pandemic. Anti-vaccination groups significantly more often experienced loss of income, loss of access to health care, and more often considered the epidemiological restrictions to be excessive. People from the anti-vaccination group obtained a significantly higher number of correct answers in the COVID-19 knowledge test.

**Table 2:** Comparison of the two groups in terms of the declared willingness to vaccinate.

	**Anti-vaccination,** ***n =* 243**	**Pro-vaccination,** ***n =* 758**	***p-*value**	***Z*-value**	**ES**
Sex (female)	182 (74.90%)	569 (75.07%)	0.976	−0.030	0.000
Age, years	38.74 ± 13.12	38.24 ± 15.08	0.252	−1.146	0.001
Place of residence (urban)	211 (86.83%)	690 (91.03%)	0.058	1.890	0.004
Education level (higher education)	175 (72.02%)	584 (77.04%)	0.143	1.464	0.002
Occupation (medical profession)	92 (37.86%)	387 (51.06%)	**<0.001**	−3.581	0.013
Chronic diseases (yes)	36 (14.81%)	174 (22.96%)	**0.004**	2.866	0.008
Psychiatric or psychological care	36 (14.81%)	136 (17.94%)	0.225	1.213	0.001
GHQ-28 positive scoring	80 (32.92%)	314 (41.42%)	**0.018**	2.280	0.005
GHQ-28—Total score	21.00 ± 12.90	23.47 ± 12.82	**0.001**	3.233	**0**.010
GHQ-28—somatic symptoms	5.08 ± 3.81	5.86 ± 3.77	**<0.001**	3.362	0.011
GHQ-28—anxiety and insomnia	5.77 ± 4.81	6.84 ± 4.60	**<0.001**	3.748	0.014
GHQ-28—social dysfunction	7.38 ± 3.07	7.75 ± 2.83	**0.032**	2.148	0.005
GHQ-28—severe depression	2.77 ± 3.56	3.02 ± 3.81	0.117	1.567	0.002
Loss of income	87 (35.80%)	190 (25.07%)	**0.001**	−3.341	0.011
Difficulty in accessing heathcare	139 (57.20%)	318 (41.95%)	**<0.001**	−4.039	0.016
Daily tracking of the epidemiological situation	68 (27.98%)	356 (46.97%)	**<0.001**	5.288	0.028
Opinion: the applied lockdown was excessive	158 (65.02%)	190 (25.07%)	**<0.001**	−11.356	0.129
Mass media as main source of information	64 (26.34%)	318 (41.95%)	**<0.001**	3.011	0.009
Knowledge about SARS-CoV-2: number of correct answers	6.83 ± 2.15	5.76 ± 2.07	**<0.001**	−6.842	0.047

*n (%) or mean ± standard deviation.*

*Data expressed as n (%) or mean (SD).*

*Significant differences (p <0.05) were marked with bold characters*.

### Logistic Regression Analysis

Table 3 shows the results of binary logistic regression. In the first model, taking into account the following GHQ-28 domains: somatic symptoms, anxiety and insomnia and social dysfunction, no factors significantly correlating with reluctance to vaccinate were found. The first model had a Negelkere's R2 coefficient of 0.015. The second model was extended over the first to include the occupation and chronic diseases. A significant negative correlation was found between the practice of a medical profession, the presence of chronic diseases and reluctance to vaccinate against SARS-CoV-2. The second model had a Negelkere's R2 coefficient of 0.037. In the third model we added the following variables: loss of income, difficult access to health care, daily monitoring of the epidemiological situation, opinion: the applied restrictions were excessive, mass media as the main source of information, and knowledge about COVID-19: number of correct answers. A significant negative relationship was found between the results of anxiety and insomnia in the GHQ-28, the practice of a medical profession, daily monitoring of the epidemiological situation, the mass media as the main source of information and reluctance to vaccinate. The following factors were found as significant positive determinants of the reluctance to vaccinate: loss of income, difficult access to health care, finding the applied lockdown as excessive and knowledge about SARS-CoV-2: number of correct answers. The third model was characterized by a definitely higher Negelkere's R2 coefficient of 0.252 as compared to the previously described models and described the effect on the dependent variable most fully.

**Table 3:** Factors related to the non-vaccination against SARS-CoV-2 using binary logistic regression analysis.

**Model (Negelkere's R2)**	**Variable**	**Beta**	**S.E**.	***p-*value**	**VIF**	**O.R**.	**95% CI**
Model 1	GHQ-28—somatic symptoms	−0.016	0.036	0.650	3.150	0.984	0.917–1.056
(0.015)	GHQ-28—anxiety and insomnia	−0.046	0.030	0.122	3.289	0.955	0.901–1.012
	GHQ-28—social dysfunction	0.013	0.035	0.706	1.741	1.013	0.946–1.085
Model 2	GHQ-28—somatic symptoms	0.001	0.037	0.984	3.209	1.001	0.931–1.075
(0.037)	GHQ-28—anxiety and insomnia	−0.053	0.030	0.080	3.300	0.948	0.894–1.006
	GHQ-28—social dysfunction	0.006	0.035	0.862	1.750	1.066	0.939–1.078
	Occupation (medical profession)	−0.450	0.180	0.012	1.007	0.638	0.448–0.907
	Chronic diseases (yes)	−0.572	0.205	0.005	1.020	0.564	0.378–0.843
Model 3	GHQ-28—social dysfunction	−0.034	0.038	0.360	1.772	0.966	0.898–1.040
(0.252)	GHQ-28—anxiety and insomnia	−0.071	0.033	0.032	3.356	0.932	0.873–0.944
	GHQ-28—somatic symptoms	0.060	0.040	0.131	3.262	1.062	0.982–1.147
	Occupation (medical profession)	−0.484	0.196	0.014	1.017	0.616	0.420–0.906
	Chronic diseases (yes)	−0.387	0.225	0.085	1.044	0.679	0.437–1.056
	Loss of income	0.359	0.177	0.043	1.027	1.431	1.012–2.025
	Difficulty in accessing health care	0.542	0.167	0.001	1.038	1.719	1.240–2.384
	Daily tracking of the epidemiological situation	−0.504	0.178	0.005	1.068	0.604	0.426–0.856
	Opinion: the applied lockdown was excessive	1.327	0.176	<0.001	1.240	3.769	2.670–5.321
	Mass media as main source of information	−0.401	0.180	0.026	1.054	0.669	0.471–0.952
	Knowledge about SARS-CoV-2: number of correct answers	0.135	0.042	0.001	1.155	1.145	1.054–1.244

*Significant associations (p < 0.05) were marked with bold characters. In parentheses below Models are given Negelkere's R2 values measuring the proportion of variance 'explained' by the regression*.

## Discussion

In this study we aimed to describe the factors influencing the decision to vaccinate against SARS-CoV-2. We observed a significantly lower severity of psychopathological symptoms measured with the GHQ-28 in people reluctant to get vaccinated compared to those considering vaccination, both in terms of the total score and all its subscales, including somatic symptoms, severe depression, social dysfunction, anxiety and insomnia. As a result of the use of binary logistic regression, it was shown that only the values in the anxiety and insomnia subscale, significantly negatively correlated with reluctance to vaccinate, turned out to be the inverse determinant of vaccination refusal.

Regarding the effect of socio-demographic variables on the decisions regarding vaccination we observed that pro-vaccination attitude was significantly more often present among medical professionals, respondents suffering from chronic diseases as well as among city dwellers and respondents with higher education level, for whom however, statistical significance was not achieved. In relation to pandemic related factors pro-vaccination attitude was more often observed among respondents who indicated daily monitoring of the epidemiological situation and more often chose the mass media as the main source of information about the pandemic. Anti-vaccination attitude was significantly more often observed in relation to the respondents who pointed to loss of income, loss of access to health care, and more often considered the epidemiological restrictions to be excessive—which factor had the highest effect size of 0.129 among bivariate variables. People from the anti-vaccination group obtained a significantly higher number of correct answers in the COVID-19 knowledge test and had the second highest effect size of 0.047.

In the survey, among more than 1,000 people, 24% of participants were willing to get vaccinated against SARS-CoV-2, 57% were unsure about vaccination and 18% were reluctant to be vaccinated. The obtained results indicate a clear polarization of the respondents in regard to the decision about vaccination. However, it is worth noting that during the distribution of the survey, reports from manufacturers detailing the efficacy and side effects of vaccines were not widely available. At that time, only the assumed mechanism of action of vaccines based on mRNA and viral vector technologies was known.

In a study by Salali and Uysal (28) 31% of the participants from Turkey and 14% from the UK were unsure whether to get the COVID-19 vaccine. In both countries, 3% of the participants refused to vaccinate. In an Italian study published in December 2020, more than three-quarters of respondents wanted the vaccine, 10% did not have a clear opinion, and only 5% said they did not want the vaccine, and 9% did not answer. Therefore, these data indicate significant differences between countries in terms of attitudes to vaccination against SARS-CoV-2 (29). Moreover, the results of our study, compared with studies from other countries carried out in the same period, indicate greater distrust of vaccines in Poland. At the time of writing this article, in autumn 2021, compared to the above-mentioned countries, Poland has a much smaller percentage of fully vaccinated people-−53%, while in Turkey it is 58%, in UK 67% and in Italy 72% (21). This observation may support the statement that the initial attitude toward SARS-CoV-2 vaccination, which we examined, did not change much under the influence of a vaccination campaign lasting almost a year and may be of key importance in understanding the causes of reluctance to vaccinate.

Almost 40% of the study participants had a high GHQ-28 score, indicating the presence of clinically significant psychopathological symptoms. These results correspond to other studies assessing the psychological burden during the COVID-19 pandemic, which is significantly greater than before the pandemic period, and moreover, it did not decrease significantly with the duration of the pandemic (16, 17). The high level of psychopathological symptoms in the study group is all the more important due to the fact that it characterized people from pro-vaccination group. In turn, reluctance to vaccinate was inversely determined by anxiety and insomnia. These results are consistent with the study by Yigit et al. (30), in which it was observed that people with high levels of anxiety of COVID-19 infection were more likely to agree to vaccination. At this point, it is worth referring to the study, where the authors, in the context of previous epidemics, described the so-called “adaptive” level of anxiety, prompting people to act prophylactically (31). According to them, this anxiety is based on a balance between excessive anxiety leading to panic inadequate to the actual threat and a complete lack of anxiety leading to ignoring the recommended preventive actions. On the other hand, when discussing the increasing anxiety in society, one should bear in mind the chronic stress theory, according to which prolonged activation of the hypothalamic-pituitary-adrenal axis negatively affects the immune system and overall health, leading to increased susceptibility to other diseases, including diseases of cardiovascular system and cancer (32). A binary logistic regression model showed that knowledge of SARS-CoV-2 is a positive determinant of anti-vaccine attitudes, which is in line with Chinese findings that greater understanding of COVID-19 does not correlate with greater vaccination propensity (33). In the study, over 90% of students declared their willingness to be vaccinated against SARS-CoV-2, while over 50% presented insufficient knowledge about the preventive behavior and symptoms of this disease. The significant difference in knowledge about COVID-19 between the anti-vaccine and pro-vaccine groups, coupled with prior observation of a lower level of anxiety in the anti-vaccine group, may indicate a potential difference in assessing the risk of infection with the virus: those who are reluctant to vaccinate may perceive the risk as lower compared to the pro-vaccine group. The sense of risk of SARS-CoV-2 varies from country to country. For example, according to the study from 2021 by Bowman et al. (34). 97% of Hong Kong respondents rated the symptoms of COVID-19 infection as serious or very serious, compared to only 20% in the UK. The higher sense of risk in Hong Kong was associated with a greater degree of hygiene and social distancing compared to the UK. In particular, almost 99% of Hong Kong respondents reported wearing a face mask, compared to 3% of the UK respondents. These results indicate the potential real impact of government policy and media information on the sense of threat and the degree of compliance with epidemiological recommendations.

The aforementioned different assessment of the risk of the SARS-CoV-2 pandemic is confirmed by the noted difference in the frequency of checking epidemiological reports in media, which may indicate emotional involvement in the course of the pandemic: belonging to the anti-vaccination group is negatively correlated with daily monitoring of the epidemiological situation. In our study, 42% of respondents monitored the epidemiological situation in the media on a daily basis. The result from the second wave of the pandemic may indicate a downward trend compared to the US study conducted during the first wave, in which 57% checked COVID-19-related news several times a day, and 84% at least once a day (34). On the one hand, this tendency can be explained by the habituation effect, and on the other hand, a greater awareness of the real risk of SARS-CoV-2, overestimated during the first wave. The obtained results show a correlation between less frequent news tracking and a lower level of anxiety. The relationship between emotional involvement and monitoring information about the epidemic is also confirmed by studies on the H1N1 (swine flu) virus epidemic, indicating a higher level of anxiety in response to greater exposure to media materials about the epidemic (35).

When analyzing the differences between groups in terms of knowledge about the pandemic, the impact of information sources on the decision to vaccinate should also be considered. Based on the binary logistic regression model, people reluctant to vaccinate against SARS-CoV-2 less frequently reported using the mass media as a source of information about the pandemic. Nekliudov et al. (36). emphasized the role of the mass media in the excessive escalation of fear related to the pandemic. On the other hand, it is worth remembering that apart from mainstream media, there are also portals where fake news and conspiracy theories are overrepresented (37). Therefore, an extended analysis of vaccination decisions in the context of infodemia is justified (38). Research indicates that 90.3% of North Americans and 61.9% of the rest of the world actively use the Internet (39). The data show that 75–80% of internet users look for health information on websites, and 70% of them say that this content influences their treatment (40). Unfortunately, the Internet still does not allow for reliable data verification, hence it is there that the fake news about pandemic and vaccines is most often spread. We can conclude that the decision to vaccinate against SARS-CoV-2 is made without verifying the information gathered by the online media (41). Interesting results were brought by the study by Salali and Uysal (28), which investigated the influence of conspiracy theories on the decision to vaccinate against SARS-CoV-2 in Great Britain and Turkey. It turned out that the belief that the pandemic started naturally had a significant impact on the pro-vaccination attitude. Another study of around 1,500 Jordanian students found higher levels of anxiety among those who believed in COVID-19 conspiracy theories compared with students who rejected them (42). A study performed by Pisl et al. (43) found that students experiencing a typical dissociative situations more often believed in conspiracy theories related to COVID-19. Believing in them might be understand as an unconscious tendency to lower the level of anxiety associated with the pandemic based on a mechanism similar to the phenomenon of dissociation. A strong long-term relationship between adherence to conspiracy theories and vaccine hesitancy (44, 45) as well as the negative impact of exposure to conspiracy theories on the willingness to vaccinate have been described (46). Bronstein et al. (47), using cutting-edge machine learning algorithms and psychometric network analysis, described a mechanism that takes into account the dependencies between tasks measuring reasoning biases, belief in conspiracy theories and reluctance to vaccinate. Reasoning biases, such as reduced data gathering related to the currently increasing tendency to stay in so-called “information bubbles” seems to be a modifiable factor leading to conspiracy believes and vaccine reluctance. It has been reported that the fear of losing a sense of control during a pandemic exacerbated the perceptions of persecution, then increased the sense of danger associated with vaccine and vaccination, and ultimately influenced the emergence of conspiracy theories. Finally reluctance to vaccinate was identified as a likely cause of belief in a conspiracy theory subverting the common assumption that the opposite causal relation exists. Unfortunately, our study did not assess belief in conspiracy theories, which should definitely be considered in further conclusions. We postulate that mental health and decision to vaccinate might be mediated by conspiracy believes regarding virus origins, vaccines and vaccination.

During the first wave of the pandemic, as in other European countries (48), the Polish government introduced the so-called total lockdown, consisting in an order to stay at home except for the necessity to meet basic life needs and go to work if it is not possible to perform it remotely (49). During the second wave, the Polish government introduced a partial lockdown, including the closure of restaurants, shopping malls, guesthouses and hotels, and recommendations for remote work were maintained (50). During the first two waves of the pandemic, wearing masks in public places, including open spaces were obligatory (51). Another explanation for such a low percentage of people willing to be vaccinated in our study may be the anti-vaccination movement in Poland. Its groups spread false information to the public, creating chaos and thus undermining confidence in the validity and safety of vaccinations. Such action causes divisions in the society and, as indicated by several authors, evokes a strong reluctance to vaccinate (52, 53).

Among the determinants of reluctance to vaccinate, the belief about excessive restrictions and the introduction of lockdown was the most important. Moreover, loss of access to healthcare and loss of income as a result of the pandemic also determined belonging to the anti-vaccine group. Such results indicate a broader aspect of the decision to vaccinate in the context of the negative impact of lockdown on the lives of citizens. Attitude toward vaccination appears to have a potential relationship to the degree of trust in the government, which imposes economic constraints, and is also involved in vaccine distribution. This hypothesis is confirmed by Italian studies conducted by Prati (29), in which the lack of intention to receive a vaccine was associated with a lower level of worry and institutional trust.

The observed ineffectiveness of lockdowns in reducing the number of SARS-CoV-2 infections, while at the same time causing the emotional burden of social isolation and economic costs should prompt governments to consider changing their strategies, especially due to the aforementioned impact of public confidence in the willingness to vaccinate against SARS-CoV-2.

Experiencing limitations and changes in many important spheres of life can cause a reaction based on the so-called defense mechanisms, e.g., denial, which in the time of a pandemic is not only to reduce the risk of infection with the virus, but also to reduce the perceived anxiety. For example, according to Johnson, “ignoring happens when an individual consciously knows that a problem exists, but chooses not to confront it” (54). Hence, there is a potential explanation that people with less severe GHQ-28 psychopathological symptoms, who are also reluctant to vaccinate, may ignore the actual situation so as not to exacerbate their anxiety.

Our study found that health care workers were less in the anti-vaccine group. These results are consistent with the studies by Akarsu et al. (55), where greater susceptibility to vaccination was also observed among medical professions. The majority of people who considered COVID-19 a very serious disease was the elderly, the chronically ill, men, people with lower incomes and lower levels of education. Therefore, it is worth considering the different social attitudes presented by the respondents at this point. People from the anti-vaccine group, due to their high knowledge of SARS-CoV-2, awareness of a relatively low risk of contracting the disease at an earlier age, no burden of chronic diseases and a lower risk of infection resulting from much less frequent work in the health service, may characterize an individualistic attitude. Focusing on your own health and the consequences of long-term lockdown restrictions can lead to opposition to vaccination as well as decisions to be made against society as a whole. In contrast, pro-vaccination people may present a collectivist attitude, characterized by respecting the common good and responsibility for the safety of the community. Our results showed that this group largely included representatives of medical professions, the elderly and people with chronic diseases, especially at risk of severe COVID-19. In the future, therefore, it is worth considering social attitudes when researching attitudes and beliefs about vaccinations.

In our study, we did not ask directly about the reasons for the reluctance to take the vaccine. In a study from Turkey, the most common reasons for refusal were concerns about the side effects of COVID-19 vaccines, a lack of knowledge about vaccine effectiveness, and distrust of vaccines from abroad (29). Similarly, in the study by Szmyd et al. (56), the desire to get vaccinated as quickly as possible was associated with lower concerns about side effects of the vaccine.

## Limitations

The strength of our study is the use of an original tool to assess the level of knowledge about COVID-19 along with the standardized GHQ-28 questionnaire to measure mental health and the assessment of sociodemographic and pandemic factors in the context of vaccination decisions. However, we do recognize some of its limitations. First, the conclusions should be generalized with caution due to the limited representativeness of the sample. We did not register the initial number of people asked to participate and we did not report the reasons for non-participation. It should also be noted that the study did not include questions about the duration of selected symptoms, hence the results relate more to short-term psychopathological episodes than to long-term mental states. It is inevitable that both the online distribution and the form of the online questionnaires themselves run the risk of bias in the responses, hence the strength of the evidence should be treated with caution. The sampling bias consists in over representing people with a special interest in the COVID-19 pandemic. As a result, our study over-represented representatives of the medical professions. Due to the online nature of the study an overrepresentation of young people and a lower representation of older people were observed. Moreover, we did not ask about the direct reason for the declared willingness or reluctance to vaccinate against SARS-CoV-2, which could provide relevant information about the motives of attitudes and decisions. Another limitation of our study was the lack of a questionnaire assessing the severity of psychotic-like experiences and a paranoid attitude, which, according to recent studies, may influence refusal of vaccination (57). It is worth noting that the GHQ-28 scale assesses the severity of symptoms such as depression and anxiety, however, it does not allow for an unequivocal psychiatric diagnosis, which should be based on a clinical examination taking into account the DSM-V or ICD-10 criteria. We also did not use other scales that would allow for the differential diagnosis of mental disorders. Finally, a significant limitation is the inability to establish a causal relationship between psychopathological symptoms, sociodemographic and pandemic factors, and between the decision to be vaccinated hence we discussed the potential impacts.

## Conclusions

Initial attitude toward SARS-CoV-2 vaccination, which we examined, may be of key importance in understanding the causes of reluctance to vaccinate. The presented study shows a significant social polarization depending on the decision to vaccinate. Greater readiness to vaccinate can be understood in terms of greater confidence in its effectiveness when a person experiences anxiety and mental deterioration, is physically burdened, is older, or is at risk of infection by working in the healthcare sector. Such an attitude may also result from relying on pro-vaccination information presented in the mass media, but also from a hypothetical collectivist attitude, in which the good of society exceeds the individual good. On the other hand, reluctance to vaccinate can be seen as greater awareness of the complexity of the disease, and thus less faith in the safety and effectiveness of vaccines. Such decisions may also be conditioned by the assessment of the pandemic situation as not so threatening and thus not causing strong symptoms of psychopathology. Resistance to vaccination is also associated with a loss of confidence in health care and the experience of loss of income, which may indicate a strict focus on one's own situation, which is explained by an individualistic attitude. More research is needed regarding the evaluation of paranoid attitudes, psychotic-like experiences and vaccination refusal. Moreover, in view of the prolonged pandemic and voluntary nature of vaccinations, longitudinal studies on representative samples are needed in order to make a reliable assessment of the long-term health and social consequences, and regarding factors contributing to vaccination decision.

## Data Availability Statement

The raw data supporting the conclusions of this article will be made available by the authors, without undue reservation.

## Ethics Statement

The studies involving human participants were reviewed and approved by Ethics Committee of the Medical University of Wroclaw (Poland, No. 188/2020). The patients/participants provided their written informed consent to participate in this study.

## Author Contributions

JM, BM, DS, and JR: conceptualization. JM, BM, and DS: methodology. JM and BM: software. WB: validation and data curation. ML-B: formal analysis. PG: investigation. JR: resources, supervision, and funding acquisition. JM, PG, and DS: writing—original draft preparation. ML-B, BM, DS, and JR: writing—review and editing. WB and PG: visualization. JM: project administration. All authors have read and agreed to the published version of the manuscript.

## Funding

This study was supported by the Wroclaw Medical University Grant (No. SUBZ.C230.22.062).

## Conflict of Interest

The authors declare that the research was conducted in the absence of any commercial or financial relationships that could be construed as a potential conflict of interest.

## Publisher's Note

All claims expressed in this article are solely those of the authors and do not necessarily represent those of their affiliated organizations, or those of the publisher, the editors and the reviewers. Any product that may be evaluated in this article, or claim that may be made by its manufacturer, is not guaranteed or endorsed by the publisher.
