# Supplementary material for: Does Mental Health Affect the Decision to Vaccinate Against SARS-CoV-2? A Cross-Sectional Nationwide Study Before the Vaccine Campaign
Source: Front Psychiatry. 2022 Feb 4;13:810529. doi: 10.3389/fpsyt.2022.810529 (PMC8854753; doi:10.3389/fpsyt.2022.810529)
Supplement: Supplementary file 1 [file Table_1.DOCX]

**Table S1.** The sociodemographic survey.

| **Questions** | **Options** |
| --- | --- |
| 1. What is your gender? | 1a. Male  1b. Female |
| 2. How old are you? | … |
| 3. What is your place of residence? | 3a. Urban  3b. Rural |
| 4. What is your education level ? | 4a. Higher education  4b. Secondary education or lower |
| 5. Are you in a medical profession? | 5a. Yes  5b. No |
| 6. Do you suffer from chronic diseases? | 6a. Yes  6b. No |
| 7. Are you receiving psychological or psychiatric care? | 7a. Yes  7b. No |
| 8. What is your attitude towards the SARS-CoV-2 vaccination? | 8a. I will definitely get vaccinated against SARS-CoV-2  8b. I would make a decision based on the ratio of vaccine effectiveness to the observed side effects  8c. I will definitely not get vaccinated against SARS-CoV-2 |
| 9. How your income changed as a result of the pandemic? | 9a. Income increased  9b. Income did not change  9c. Income decreased |
| 10. How do you rate access to healthcare during the pandemic? | 10a. Easy access  10b. Moderate access  10c. Difficult access |
| 11. How often do you monitor the epidemiological situation? | 11a. Every day  11b. Once every few days  11c. Less often than once every few days |
| 12. How do you rate the extent of the previously introduced lockdown? | 12a. Lockdown has been implemented to a reasonable extent  12b. Implemented lockdown has been insufficient  12c. Implemented lockdown has been excessive |
| 13. What is your main source of knowledge about the COVID-19 pandemic? | 13a. Mainstream media  13b. Medical publications  13c. Other sources |
