# Supplementary material for: Does Mental Health Affect the Decision to Vaccinate Against SARS-CoV-2? A Cross-Sectional Nationwide Study Before the Vaccine Campaign
Source: Front Psychiatry. 2022 Feb 4;13:810529. doi: 10.3389/fpsyt.2022.810529 (PMC8854753; doi:10.3389/fpsyt.2022.810529)
Supplement: Supplementary file 2 [file Table_2.DOCX]

**Table S2.** Questionnaire of knowledge about the SARS-COV-2 pandemic.

| **Questions** | **Options** |
| --- | --- |
| 1. Indicate the current definition of a pandemic according to the World Health Organization (WHO) | **1a. Significantly increased number of cases over a large area over a period of time (n=447, 44.6%)**  1b. Significantly increased mortality over a large area over time (n=13, 1.3%)  1c. Significantly increased number of cases and significantly increased mortality over a large area over a given period of time (n=542, 54.1%) |
| 2. What is the real mortality rate of the SARS-COV-2 in Poland in the general population? | 2a. over 5% (n=23, 2.3%)  2b. between 3-5% (n=259, 25.9%)  **2c. less than 3% (n=720, 71.9%)** |
| 3. What percentage of people who test positive for SARS-COV-2 have an infection that is asymptomatic or mildly symptomatic? | **3a. over 75% (n=490, 48.9%)**  3b. between 50% and 75% (n=397, 39.6%)  3c. less than 50% (n=115, 11.5%) |
| 4. What is the average age of patients who died due to SARS-COV-2 infection? | **4a. over 70 years old (n=588, 58.7%)**  4b. between 50 and 70 years old (n=406, 40.5%)  4c. under 50 years old (n=8, 0.8%) |
| 5. How has the total monthly number of deaths in Poland changed during the first months of the pandemic in March and April 2020 compared to the corresponding months in 2018 and 2019? | 5a. The total number of deaths increased by over 30% (n=47, 4.7%)  5b. The total number of deaths increased by 15-30% (n=312, 31.1%)  **5c. The total number of deaths has decreased or has not changed significantly (n=643, 64.2%)** |
| 6. What percentage of patients who died from SARS-COV-2 virus had other comorbidities? | **6a. Above 70% (n=843, 84.1%)**  6b. Between 30-70% (n=143, 14.3%)  6c. Less than 30% (n=16, 1.6%) |
| 7. What percentage of daily deaths in Poland at the peak of the first spring wave of the pandemic (March, April 2020) were deaths due to COVID-19? | 7a. Above 15% (n=48, 4.8%)  7b. Between 5 and 15% (n=241, 24.1%)  **7c. Less than 5% (n=713, 71.2%)** |
| 8. What percentage of hospital beds in wards dedicated to COVID-19 patients at the peak of the first spring wave of the pandemic (March, April 2020) were occupied by patients with SARS-COV-2 infection in Poland? | 8a. Above 70% (n=131, 13.1%)  8b. Between 30 and 70% (n=392, 39.1%)  **8c. Less than 30% (n=479, 47.8%)** |
| 9. What is the degree of protection of a person wearing a cloth mask against infection with SARS-COV-2? | 9a. Completely protects the wearer (n=20, 2.0%)  9b. It protects the wearer significantly (n=525, 52.4%)  **9c. Practically no protection for the wearer (n=457, 45.6%)** |
| 10. After what time should the cloth or surgical mask be changed or washed to avoid the development of bacterial colonies on its surface? | 10a. Every 1 day (n=128, 12.8%)  10b. Every few hours (n=221, 22.1%)  **10c. Not less than every hour (n=653, 65.2%)** |

Data expressed as n (% ) of the total sample. Correct answers are marked with bold characters.
